# Development and Validation of the Digital Sensitivity Scale for Adults: Cross-Sectional Observational Study

**DOI:** 10.2196/55828

**Published:** 2025-01-10

**Authors:** Hae In Park, Minjeong Jeon, Ji Seon Ahn, Kyungmi Chung, Jin Young Park

**Affiliations:** 1 Department of Psychiatry Yongin Severance Hospital Yongin Republic of Korea; 2 Department of Psychiatry Yonsei University College of Medicine Seoul Republic of Korea; 3 Institute of Behavioral Science in Medicine Yonsei University College of Medicine Seoul Republic of Korea; 4 Center for Digital Health Yongin Severance Hospital Yonsei University Yongin Republic of Korea

**Keywords:** information literacy, health literacy, computer literacy, self-efficacy, attitude, digital divide

## Abstract

**Background:**

The COVID-19 pandemic has accelerated the digitalization of modern society, extending digital transformation to daily life and psychological evaluation and treatment. However, the development of competencies and literacy in handling digital technology has not kept pace, resulting in a significant disparity among individuals. Existing measurements of digital literacy were developed before widespread information and communications technology device adoption, mainly focusing on one’s perceptions of their proficiency and the utility of device operation. In the contemporary landscape, digital transformation is evolving within specialized domains, necessitating a comprehensive evaluation of digital competencies, attitudes, and proficiency in technology application to bridge the digital divide and ensure digital compliance.

**Objective:**

This study was designed to address the shortcomings of existing scales and formulate a digital sensitivity scale tailored to the requirements of today’s society.

**Methods:**

Initial items of the Yongin Severance Digital Sensitivity Scale (YI-DSS) were collected through a literature review, and expert opinions were gathered to ensure content validity. An exploratory and confirmatory factor analysis included 986 adult participants evaluating 14 digital literacy items and 6 digital efficacy items. The Cronbach α confirmed internal consistency reliability, and 2-tailed *t* tests, ANOVAs, and post hoc tests analyzed demographic differences in digital literacy and efficacy.

**Results:**

A robust 4-factor digital literacy solution was identified: digital application, digital communication, critical thinking, and digital ethics (Kaiser-Meyer-Olkin=0.891; Bartlett × 2=9829.713; *P*<.001; Cronbach α=0.782-0.947). A 2-factor solution defined digital efficacy: digital confidence and digital anxiety (Kaiser-Meyer-Olkin=0.735; Bartlett × 2=3282.217; *P*<.001; Cronbach α=0.787-0.912). Confirmatory factor analysis was conducted for each model (digital literacy model: *χ*^2^_71_=676.0, comparative fit index=0.938, Tucker-Lewis index=0.921, standardized root mean square residual=0.73, and root mean square error of approximation=0.093; digital efficacy model: *χ*^2^_8_=81.9, comparative fit index=0.977, Tucker-Lewis index=0.958, standardized root mean square residual=0.73, and root mean square error of approximation=0.097), which indicated a good fit. The YI-DSS also showed high correlation with the previously developed Digital Literacy Scale (*r*=0.809; *P*<.001).

**Conclusions:**

The YI-DSS, as a self-assessment tool, has the potential to bridge the generational information gap by promoting acceptance, motivation, and adaptation to digital technology. Furthermore, given the remote nature of digital therapeutics, an individual’s familiarity with required technologies and digital communication strongly influences their acceptance of digital treatments and the efficacy thereof. This scale can play a pivotal role in enhancing compliance with digital therapeutics by preemptively assessing individuals’ technological literacy and competency.

## Introduction

### Background

The COVID-19 pandemic has accelerated digitalization in various sectors, with a notable impact in the field of psychiatry. This shift was essential in maintaining psychological evaluations and treatments during physical distancing, demonstrating the effectiveness of digital approaches in improving mental health care. The incorporation of technology into everyday clinical practice has transitioned from being just a necessity to a vital element of psychiatric services. Despite the successful application of digital therapeutics in treating various conditions such as insomnia, attention-deficit/hyperactivity disorder in children, and substance use disorder [1-4], there are significant barriers that hinder their widespread adoption. In a comprehensive review conducted by van Kessel et al [5], the key factors influencing the assimilation of digital therapeutics into health care systems and their practical application by patients and professionals were examined, identifying digital literacy as the most critical factor according to health care professionals. Specifically, they highlighted a significant concern: if the design of digital therapeutics fails to consider the potential exclusion of individuals with limited digital literacy, it could inadvertently aggravate existing disparities in health care access and outcomes. Therefore, given its pivotal role in the assimilation and practical application of digital therapeutics, digital literacy emerges as a crucial element in the field.

Digital literacy, recognized as a key concept within digital competence, was first introduced by Gilster [6], and its conceptual framework has undergone progressive refinement over time. According to Gilster [6], digital literacy goes beyond the ability to simply operate a computer; it refers to the ability to evaluate and judge digitized information (content evaluation) and combine the collected information with new information to achieve specific personal goals (knowledge), thereby ensuring the proper use of information. This concept has evolved into an essential survival skill essential to the members of modern society, and it is presented as a concept that includes the values of digital citizenship, such as social participation and moral attitude in the digital environment [7-11]. In short, digital literacy is a concept that encompasses the cognitive ability to access and understand digital information and the ability to create new and alternative content, as well as the moral responsibility of communicating in a digital environment and solving problems ethically.

Digital literacy is a comprehensive framework for the complex and integrated subfields of technology, knowledge, ethics, and creative production in a digital network environment. In the *Digital literacy across the curriculum* handbook developed for education workers, Futurelab in the United Kingdom presented eight components of digital literacy: (1) functional skills in information and communications technology (ICT), (2) creativity in developing new content and results, (3) collaboration, (4) effective communication using digital technology, (5) the ability to find and select digital information, (6) critical thinking and evaluation, (7) cultural and social understanding, and (8) e-safety in using digital information without violating laws and ethics [12]. Eshet-Alkalai and Chajut [13] proposed six concepts constituting a framework for digital literacy: (1) photovisual literacy, which refers to the ability to work effectively in interfaces such as graphic communication; (2) reproduction literacy, which refers to the ability to manipulate existing digital information to create meaningful new content; (3) information literacy, which refers to the ability to critically consume information; (4) branching literacy, which refers to the ability to construct knowledge through nonlinear search; (5) socioemotional literacy, which refers to the ability to communicate effectively in online communication spaces; and (6) real-time thinking technique, which refers to the ability to process and evaluate large amounts of information in real time.

According to a report by the Korea Education and Research Information Service (2017), in South Korea, the domains of digital literacy are (1) understanding and using digital technologies, (2) digital consciousness and attitudes, (3) digital thinking skills, and (4) digital practice competence. The study by Kang et al [14] on digital literacy measurement in a smart society suggested that there are 3 domains of diagnostic indexes for digital literacy measurement: technology, application, and mind. The technology domain measured the technological application of ICT, and the application domain measured whether digital technology and knowledge are applied to life. The mind domain in particular measured information ethics awareness and norms required in a smart society, which was different from the finding of previous studies that focused on ICT’s instrumental ability and technological proficiency. In his study on digital capabilities in the Fourth Industrial Revolution era, Choi [15] presented the five domains of the framework for digital competence: (1) the understanding of digital society and digital citizenship, (2) communication and collaboration using digital technologies, (3) critical thinking and information literacy, (4) computing thinking and problem-solving, and (5) creative and convergence thinking and content creation.

This proposition was meaningful because it emphasized not only digital citizenship and communication skills and ethics in the digital world, such as the impact of digital technology on society, but also computational thinking, such as programming and modeling. It was also noteworthy that the study used the term “digital competence,” emphasizing that “competence” refers to the ability to apply knowledge, expertise, and skills required for effective performance in specific situations. This usage was considered more appropriate than “literacy,” which primarily focuses on the encoding and decoding of information [15]. Shin and Lee [16] measured the digital literacy of college students required in a software-oriented society by presenting four factors: (1) basic competence in ICT, (2) software-oriented social adaptation ability, (3) social networking service (SNS) use and collaboration ability, and (4) basic work use ability, where software-based communication and collaboration capabilities were reflected in addition to competence in ICT use.

Although various previous studies, including the ones mentioned previously, deal with and measure the characteristics and components of digital literacy, they have some limitations. Existing scales simply focus on the operational capability of ICT equipment without incorporating ethical values such as correct information ethics, information use attitude, sound communication, and citizenship [17,18]. Furthermore, many studies were either conducted before the popularization of ICT devices or targeted teachers or students for the purpose of promoting digital literacy education. With the emergence of various digital devices, media, and technologies, there is an increasing need to measure literacy in this new environment.

Meanwhile, studies have suggested 3 stages in the development of digital literacy: digital competence, digital use, and digital transformation [19]. The first stage, digital competence, refers to the confident and critical use of ICT for work, leisure, or communication; it consists of basic knowledge, skills, and attitudes about ICT. Individuals successfully use digital competence for their circumstances and apply it to various situations and contexts to achieve various goals. The second stage, digital use, refers to the application of digital competence to specific situations, including the use of digital tools to solve problems or develop problem-solving methods and to share and learn information within a community of practice. In the final stage, digital transformation, the advances in digital use bring innovation and creativity, effecting significant changes in areas of expertise or knowledge. Although each step may not be performed sequentially, in many cases, skills or knowledge of the digital competence level may be required to achieve innovation at a higher level, such as digital transformation. For this developmental process to occur smoothly, intrinsic factors such as interest and motivation that enable the acceptance and use of digital technologies are necessary [19]. This study intended to present self-efficacy as a personal characteristic that is expected to influence the use of digital technology.

Self-efficacy refers to the belief in one’s ability to execute and organize the course of action required to achieve a particular outcome [20]. Individuals’ self-efficacy affects their ability to undertake tasks and accept challenges, and it is applicable to various situations [21]. For example, changing the way in which education is delivered—from face-to-face education to non–face-to-face education through computers—may affect students’ sense of computer-using self-efficacy [22,23]. Students with previous computer or online training reported a higher level of computer self-efficacy [23,24]. With a high level of computer self-efficacy, the students found it easier to engage in online learning and, thus, participated in online learning for a longer time [25]. In other words, self-efficacy in the digital environment is expected to affect individuals’ use of digital technology.

### Objectives

This study aimed to develop and present a digital literacy scale suitable for current society while also addressing the limitations of existing scales. In this study, *digital technology literacy* refers to the technical ability to properly handle information and communication equipment (eg, computers, smartphones, and tablets) and the various abilities required to perform tasks in a digital environment. The components of digital technology literacy reconstructed in this study based on the concept of digital literacy presented in previous studies are as follows: (1) the ability to create and reproduce new content based on digital technology, (2) the ability to communicate and collaborate to solve problems using digital technology, (3) the ability to verify the reliability and accuracy of digital information and use it critically, and (4) the ability to comply with ethical guidelines and understand ethical issues that arise from using digital technology. Furthermore, to measure personal factors that may influence the use of digital technology, this study also intended to measure the sense of efficacy in using digital technology. Previous research indicates that self-efficacy levels influence technology use, so this study integrated elements of digital efficacy along with digital literacy. Moreover, to gauge digital literacy across a wide age range, we planned to assess from individuals in their 20s to those aged ≥60 years.

## Methods

### Preliminary Item Development

For the development of the Yongin Severance Digital Sensitivity Scale (YI-DSS), 6 factors were derived through literature review, with 3 to 4 items being developed for each factor, accounting for a total of 20 items. To check the suitability of the developed items for measuring digital literacy and digital efficacy, the goodness of fit was verified by experts to ensure content validity. The evaluation was conducted by 20 experts, including professors in engineering, psychology, or medicine or experts who had worked in the field for >10 years, and the data collected from 18 experts were used for analysis.

### Study Design

This was an observational, cross-sectional study conducted through an online survey. The survey was administered to members of a survey site, and data were collected between May 2023 and July 2023. This study adheres to the Checklist for Reporting Results of Internet E-Surveys guidelines for reporting internet survey results [26].

### Participants

An online survey was conducted with 1000 adults aged ≥19 years. Participants eligible for inclusion in the study were adults aged ≥19 years, with quotas set to ensure equal distribution by age and gender and proportional distribution by region. Exclusion criteria included individuals who did not complete the survey or provided responses that appeared insincere based on response patterns. The results were obtained based on the responses of 986 participants, excluding the insincere responses of 14 participants.

### Setting

This study recruited a sample from a web panel of individuals registered with Macromill Embrain, a professional survey company specializing in web-based survey and research services. Macromill Embrain maintains a diverse participant pool through continuous recruitment efforts across various demographics (eg, age, gender, and region) and identity verification processes. For this study, participants aged ≥19 years residing in South Korea were selected using quota sampling. Age group and gender were equally allocated, whereas region was proportionally allocated based on the population distribution across the country’s 17 major provinces. Participants who completed the consent process were asked to answer screening questions on eligibility and basic demographic information. Those who met the eligibility criteria were invited to participate in the main survey, whereas those who did not qualify or exceeded the required quotas received compensation according to Embrain’s policy. Information about this compensation was provided at the bottom of the initial survey screen. Eligible participants were then randomly selected from the assigned quota groups and invited through a link containing a brief description of the study and eligibility criteria.

Participation in the survey was entirely voluntary, and participants could withdraw at any time before submitting their responses without penalty. They were also able to review and change their answers before the final submission, ensuring flexibility. Survey responses were automatically recorded and securely stored in a database. Only completed questionnaires were analyzed.

### Sample Size Calculation

According to absolute criteria in factor analysis, a sample size of 100 is considered poor, a sample size of approximately 200 is fair, a sample size of approximately 300 is good, a sample size of approximately 500 is very good, and a sample size of ≥1000 is excellent [27]. In terms of the ratio of sample size to the number of measured variables, various scholars suggest different ratios, but it is generally recommended to have a sample size that is at least 20 times the number of factors to be extracted to obtain stable factors [28]. Considering the aforementioned criteria, the total target number of participants was set at 1000.

### Statistical Analysis

The data collected were statistically analyzed using SPSS (version 27.0; IBM Corp) and R (R Foundation for Statistical Computing). First, exploratory factor analysis was performed to extract the factors of the YI-DSS. Items with communality values of <0.4 were considered unsuitable for the factor structure and were removed before conducting the analysis. To examine whether the collected data were suitable for analysis, the Kaiser-Meyer-Olkin (KMO) test for sampling adequacy and Bartlett test of identity matrix were performed. Principal component analysis was performed for exploratory factor analysis, and the varimax rotation was used for factor rotation. Second, confirmatory factor analysis was performed to verify whether the items were properly set in the constructs derived through exploratory factor analysis. Third, the Pearson correlation coefficient was used to identify the concurrent validity of the Digital Literacy Scale developed in the previous study. Fourth, the Cronbach α was obtained to verify the internal consistency reliability of the scale. Finally, the differences in digital literacy and digital efficacy according to demographic characteristics were analyzed using 2-tailed *t* tests and ANOVAs. For cases in which significant differences between groups were found, the Scheffé post hoc analysis was conducted. When heteroscedasticity was identified in the test for homogeneity of variances, the Dunnett T3 test, which accounts for unequal variances, was performed.

Data cleaning, exploratory factor analysis, correlation coefficient analysis, *t* tests, and ANOVAs were conducted using SPSS (version 27.0). Confirmatory factor analysis was performed using the *lavaan* package in R.

### Ethical Considerations

This study was approved by the institutional review board of Yongin Severance Hospital (9-2022-0199). Before obtaining consent to participate in the study, the purpose and procedures of the research were explained, including the voluntary nature of participation and the right to withdraw at any time. All personal information provided by participants was anonymized, encrypted, and securely managed. In accordance with the Bioethics and Safety Act, the data will be destroyed 3 years after the conclusion of the study. Monetary incentives were offered to participants upon survey completion to encourage participation.

### Measure and Scale

For the development of the YI-DSS, the concepts and components of digital literacy and digital efficacy were defined, as shown in Table 1. For digital literacy, 4 factors—digital application, digital communication, critical thinking, and digital ethics—were defined, and for digital efficacy, 2 factors—digital confidence and digital anxiety—were established.

Preliminary items were created for the development of a scale that reflected the concepts of the components to assess digital literacy and digital efficacy. In total, 3 to 4 items were developed for each construct, and a total of 20 preliminary items were selected. Each item was scored on a 7-point Likert scale, from 1 point for “strongly disagree” to 7 points for “strongly agree”—a higher score indicates higher digital literacy. Table 2 shows the selected items.

**Table 1:** Components of the scale.

Component		Definition
**Digital literacy**		It refers to the competence to critically understand digital information using ICT^a^ equipment and digital technology and create new content through technology convergence to solve problems cooperatively.
	Digital application	The ability to create and reproduce new content based on digital technology
	Digital communication	The ability to communicate and collaborate to solve problems using digital technology
	Critical thinking	The ability to verify the reliability and accuracy of digital information and use it critically
	Digital ethics	The ability to comply with ethical guidelines and understand ethical issues that arise from using digital technology
**Digital efficacy**		It refers to the sense of having the competence to embrace digital technology and confidently apply it to solve everyday problems.
	Digital confidence	The confidence to use digital technology to solve problems
	Digital anxiety	Distrust, concern, and anxiety about the use of digital technology

^a^ICT: information and communications technology.

**Table 2:** Items for the development of the scale.

Construct and item ID			Content
**Digital application**			
	DA1	“I can use digital technologies to create new content.”	
	DA2	“I create my own content to convey information accurately.”	
	DA3	“I am skilled in producing digital content in various formats.”	
	DA4	“Producing content directly using digital technology is not challenging for me.”	
**Digital communication**			
	DCom1	“When I acquire new information, I share it in online spaces.”	
	DCom2	“I find joy in engaging in online conversations and listening to others.”	
	DCom3	“I actively express my views in online communities through posts, comments, etc., or participate in and signing petitions.”	
**Critical thinking**			
	CT1	“Before utilizing information I’ve searched for, I verify its accuracy and reliability.”	
	CT2	“I read information from multiple web sources and cross-check its accuracy before using it.”	
	CT3	“Before using online information, I ensure it is up-to-date.”	
	CT4	“Before utilizing online information, I verify the reliability of the information provider.”	
**Digital ethics**			
	DE1	“I do not leak personal information about others or upload or download photos or videos of others without their permission.”	
	DE2	“I ethically source information on the web and utilize it directly from its original source.”	
	DE3	“I do not create or share materials online that are harmful to others or illegal.”	
**Digital confidence**			
	DCon1	“I effectively utilize digital information.”	
	DCon2	“I am confident in utilizing digital technology.”	
	DCon3	“I believe I can solve a problem that arises while using digital technologies myself.”	
**Digital anxiety**			
	DAnx1	“I feel unsafe using digital technologies as I am concerned about device malfunctions, loss of information, hacking, etc.”	
	DAnx2	“I am hesitant to use digital technologies as I am not confident in using them.”	
	DAnx3	“I am afraid of accidentally losing information while using digital technologies.”	

The concurrent validity between the YI-DSS developed in this study and the previously developed Digital Literacy Scale was confirmed. The Digital Literacy Scale is a self-report scale developed by Lim et al [29] and consists of a total of 24 questions in 5 factors (using digital information and data, creating and expressing digital content, digital communication and collaboration, information protection and compliance with laws and regulations, and computing thinking). The construct validity of the scale was confirmed through exploratory factor analysis and confirmatory factor analysis, and the internal reliability of the scale was found to be 0.941. The responses are rated on a 5-point Likert-type scale.

## Results

### Demographics

Table 3 presents data on the gender, age, and educational background of the respondents to the questionnaire used to obtain the study results.

**Table 3:** Demographics (N=986).

Characteristic		Participants, n (%)
**Gender**		
	Male	499 (50.6)
	Female	487 (49.4)
**Age (y)**		
	20-29	159 (16.1)
	30-39	164 (16.6)
	40-49	200 (20.3)
	50-59	210 (21.3)
	60-64	103 (10.4)
	>65	150 (15.2)
**Educational level**		
	Elementary school graduate	1 (0.1)
	Middle school graduate	9 (0.9)
	High school graduate	205 (20.8)
	Graduate	617 (62.6)
	Master’s degree	78 (7.9)
	Doctorate degree	29 (2.9)

### Factor Analysis

Table 4 presents the results of the exploratory factor analysis conducted on the Digital Literacy Scale.

All 20 items met the standard (>0.5) in the measure of communality. The results of the KMO and Bartlett test of sphericity were confirmed. A KMO metric value of ≥0.90 was considered excellent, and a value of ≤0.50 was considered unacceptable. In this analysis, the KMO value of the Digital Literacy Scale was found to be good at 0.891, and the Bartlett sphericity was also statistically significant (χ^2^_91_=9829.7; *P*<.001), indicating that the collected data were suitable for factor analysis.

Regarding each domain, digital application consisted of 4 items, with an eigenvalue of 6.289 and a variance explanatory power of 25.56%. The critical thinking factor consisted of 4 items, with an eigenvalue of 2.552 and a variance explanatory power of 21.02%. The digital ethics factor consisted of 3 items, with an eigenvalue of 1.035 and a variance explanatory power of 15.84%. The digital communication factor consisted of 3 items, with an eigenvalue of 1.013 and a variance explanatory power of 15.363%. Finally, the total variance explanatory power of the 4 factors of digital literacy was confirmed to be 77.782%.

The internal consistency (Cronbach α) of each factor in the Digital Literacy Scale was analyzed. Table 5 shows the results. The Cronbach α was 0.974 for digital application, 0.889 for critical thinking, 0.806 for digital ethics, and 0.782 for digital communication, with the overall Cronbach α for the Digital Literacy Scale being 0.900, indicating a desirable level of reliability for all factors and the scale.

**Table 4:** Exploratory factor analysis on the Digital Literacy Scale (DLS)^a^.

Item ID	Communality	DLS			
		Digital application	Critical thinking	Digital ethics	Digital communication
DA1	0.869	0.872	0.256	0.031	0.204
DA3	0.909	0.904	0.227	0.007	0.203
DA2	0.831	0.838	0.147	–0.027	0.326
DA4	0.847	0.874	0.173	0.004	0.232
CT1	0.622	0.414	0.628	0.159	0.180
CT3	0.800	0.178	0.828	0.242	0.156
CT4	0.848	0.181	0.860	0.180	0.207
CT2	0.784	0.185	0.809	0.252	0.176
AT2	0.814	–0.045	0.098	0.896	–0.005
AT1	0.678	0.143	0.351	0.722	0.117
AT3	0.731	–0.056	0.228	0.820	0.065
DCom1	0.746	0.296	0.248	–0.050	0.771
DCom2	0.810	0.208	0.191	0.064	0.852
DCom3	0.601	0.332	0.133	0.185	0.662
Eigenvalue	—^b^	6.289	2.552	1.035	1.013
Variance explanatory power (%)	—	25.56	21.02	15.84	15.363
Cumulative variance explanatory power (%)	—	25.56	46.58	62.42	77.782

^a^Kaiser-Meyer-Olkin=0.891; Bartlett × 2=9829.713; *P*<.001.

^b^Not applicable.

**Table 5:** Subfactors of the Digital Literacy Scale and their reliability.

	Item ID	Items, N	Cronbach α
Digital application	DA1, DA2, DA3, and DA4	4	0.974
Critical thinking	CT1, CT2, CT3, and CT4	4	0.889
Digital ethics	DE1, DE2, and DE3	3	0.806
Digital communication	DCom1, DCom2, and DCom3	3	0.782
Digital Literacy Scale: total	—^a^	14	0.900

^a^Not applicable.

A total of 6 preliminary items were selected through literature review and a suitability survey by experts to measure the attitude toward using digital technology in the Digital Efficacy Scale. Each item was scored on a 7-point Likert scale from 1 point for “strongly disagree” to 7 points for “strongly agree.” Exploratory factor analysis was performed to extract the factors for the Digital Efficacy Scale. Table 6 shows the results.

In this analysis, the KMO value of the Digital Efficacy Scale was found to be acceptable at 0.734, and the Bartlett sphericity was also statistically significant (χ^2^=3389.5; *P*<.001), indicating that the collected data were suitable for factor analysis.

Regarding each domain, the digital confidence factor consisted of 3 items, with an eigenvalue of 2.651 and a variance explanatory power of 44.181%. The digital anxiety factor consisted of 3 items, with an eigenvalue of 2.112 and a variance explanatory power of 35.197%. Finally, the total variance explanatory power of the 2 factors of digital efficacy was confirmed to be 79.378%.

The internal consistency (Cronbach α) of each factor in the Digital Efficacy Scale was analyzed. Table 7 shows the results. The Cronbach α was 0.912 for digital confidence and 0.787 for digital anxiety, with the overall Cronbach α for the Digital Efficacy Scale being 0.720, indicating an acceptable level of reliability for all factors and the scale.

Following the analysis of the exploratory factor analysis results, confirmatory factor analysis of the digital literacy and digital efficacy models was conducted using the *lavaan* package in RStudio (Posit PBC). To evaluate the model fit, several indexes were computed, including the chi-square test, Tucker-Lewis index (TLI), comparative fit index (CFI), standardized root mean square residual (SRMR), and root mean square error of approximation (RMSEA). TLI and CFI values of >0.90 indicate a good model fit, whereas SRMR values of ≤0.08 and RMSEA values of <0.05 suggest a good fit.

As shown in Table 8, neither the digital literacy nor the digital efficacy models demonstrated good fit based on the chi-square statistic (digital literacy model: χ^2^_71_=676.0 and *P*<.001; digital efficacy model: χ^2^_8_=81.9 and *P*<.001). However, it is well known that the chi-square statistic is highly sensitive to sample size, so it is important to consider other fit indexes as well. The CFI, TLI, SRMR, and RMSEA values all indicated a good fit, supporting the acceptability of each model’s fit.

**Table 6:** Exploratory factor analysis on the Digital Efficacy Scale (DES)^a^.

Item ID	Communality	DES	
		Digital confidence	Digital anxiety
DCon1	0.862	0.926	–0.069
DCon2	0.885	0.937	–0.090
DCon3	0.803	0.894	–0.054
DAnx2	0.653	0.179	0.788
DAnx1	0.767	–0.227	0.846
DAnx3	0.792	–0.181	0.871
Eigenvalue	—^b^	2.651	2.112
Variance explanatory power (%)	—	44.181	35.197
Cumulative variance explanatory power (%)	—	44.181	79.378

^a^Kaiser-Meyer-Olkin=0.734; Bartlett × 2=3389.500; *P*<.001.

^b^Not applicable.

**Table 7:** Subfactors of the Digital Efficacy Scale and their reliability.

	Item ID	Items, N	Cronbach α
Digital confidence	DCon1, DCon2, and DCon3	3	0.912
Digital anxiety	DAnx1, DAnx2, and DAnx3	3	0.787
Digital Efficacy Scale: total	—^a^	6	0.720

^a^Not applicable.

**Table 8:** Goodness-of-fit indexes for the confirmatory factor analysis models.

Model	Chi-square (*df*)	*P* value	CFI^a^	TLI^b^	SRMR^c^	RMSEA^d^
Digital literacy	676.0 (71)	<.001	0.938	0.921	0.073	0.093
Digital efficacy	81.9 (8)	<.001	0.977	0.958	0.073	0.097

^a^CFI: comparative fit index.

^b^TLI: Tucker-Lewis index.

^c^SRMR: standardized root mean square residual.

^d^RMSEA: root mean square error of approximation.

### Covalidation Analysis

Pearson correlation coefficient analysis was conducted to test the covalidation between the YI-DSS and the previously developed Digital Literacy Scale. The total scores on the YI-DSS showed high correlation with the existing Digital Literacy Scale (*r*=0.809; *P*<.001).

### Further Analysis

Analysis of digital device use by age group revealed that smartphone use frequency was notably high among all participants (Table 9). Of the 986 participants, 968 (98.2%) reported using a smartphone either “frequently” or “very frequently.” Regarding PC use, 78.2% (771/986) of the participants reported frequent use, with approximately 70% of those aged ≥65 years (103/150, 68.7%) also reporting frequent use. In the case of digital communication tools, >73% of the respondents reported frequent use of both SNSs (748/986, 75.9%) and email (720/986, 73%). The use frequency of emerging technologies such as virtual reality, 3D printers (81/986, 8.2%), and voice assistants was low, with <17% (164/986, 16.6%).

An independent-sample *t* test was conducted to examine the difference in digital sensitivity scores by gender (Table 10). The Levene test was used to assess the equality of variances, and the *t* test results were presented based on whether equal variances were assumed. The analysis revealed significant gender differences in both the total digital literacy score and the total digital efficacy score.

In detail, men (mean 66.735, SD 13.008) scored significantly higher than women (mean 62.834, SD 13.775) on total digital literacy (t_984_=4.574; *P*<.001). Given the significant gender difference in the total digital literacy score, further analysis was conducted on the subfactors. The results indicated significant gender differences across all subfactors. Men scored significantly higher than women in digital application (mean 15.543, SD 5.847 for men vs mean 12.803, SD 6.158 for women; t_984_=7.167; *P*<.001). Similarly, in the critical thinking factor, men (mean 20.764, SD 4.242) outperformed women (mean 19.762, SD 4.887; t_984_=–2.658; *P*<.01). The digital communication scores were also significantly higher for men (mean 13.527, SD 3.692) than for women (mean 12.799, SD 3.859; t_984_=3.029; *P*=.003). Conversely, in the digital ethics factor, women (mean 17.470, SD 3.536) scored significantly higher than men (mean 16.902, SD 3.536; t_984_=–2.658; *P*=.008).

Regarding digital efficacy, men (mean 25.363, SD 5.152) also scored higher than women (mean 23.895, SD 4.713; t_984_=4.664; *P*<.001). Analyzing the subfactors, men showed significantly higher scores in digital confidence (mean 14.719, SD 3.656 for men vs mean 12.669, SD 4.153 for women; *P*<.001). However, in the digital anxiety factor, women (mean 11.226, SD 3.543) scored significantly higher than men (mean 10.643, SD 3.997; t_984_=–2.423; *P*=.02).

To examine whether there were differences in average digital sensitivity across different age groups, a 1-way ANOVA was conducted. A statistically significant difference was found in the total digital literacy score (*F*_5_=18.076; *P*<.001) based on a significance level of .001. Post hoc analysis revealed significant differences in the average total digital literacy scores between individuals in their 20s and 30s and those in their 40s, 50s, and 60s.

The analysis of differences across subfactors by age group is as follows. In the digital application subfactor, individuals in their 20s scored higher than those in their 40s, 50s, and 60s. Individuals in their 30s also scored significantly higher than those in their 50s and 60s, whereas those in their 40s scored higher than those in their 60s. This indicates that individuals in their 60s had the lowest ability to produce information using digital technologies compared to other age groups. In the critical thinking subfactor, individuals in their 20s and 30s scored higher than those in their 50s and 60s, and individuals in their 40s also showed significant differences compared to those in their 50s and 60s. This suggests that individuals in their 60s had a lower ability to critically use digital information compared to other age groups except for those in their 50s. In the digital communication subfactor, individuals in their 20s scored higher than those in their 60s in terms of using digital technologies for communication. Finally, in the digital ethics subfactor, there were no significant differences between age groups.

**Table 9:** Frequency of digital technology use by age group (N=986).

Type of digital technology and age group (y)		Use frequency among participants, n (%)				
		Never	Rarely	Occasionally	Frequently	Very frequently
**Digital device: PC (desktop or laptop)**						
	20-29	0 (0)	5 (0.5)	16 (1.6)	41 (4.2)	97 (9.8)
	30-39	0 (0)	6 (0.6)	20 (2)	32 (3.2)	106 (10.8)
	40-49	0 (0)	10 (1)	22 (2.2)	44 (4.5)	124 (12.6)
	50-59	0 (0)	15 (1.5)	28 (2.8)	62 (6.3)	105 (10.6)
	60-64	0 (0)	9 (0.9)	17 (1.7)	32 (3.2)	45 (4.6)
	>65	0 (0)	14 (1.4)	33 (3.3)	55 (5.6)	48 (4.9)
**Digital device: smartphone**						
	20-29	0 (0)	0 (0)	0 (0)	9 (0.9)	150 (15.2)
	30-39	0 (0)	1 (0.1)	0 (0)	23 (2.3)	140 (14.2)
	40-49	0 (0)	0 (0)	2 (0.2)	34 (3.4)	164 (16.6)
	50-59	0 (0)	1 (0.1)	8 (0.8)	40 (4.1)	161 (16.3)
	60-64	0 (0)	0 (0)	3 (0.3)	23 (2.3)	77 (7.8)
	>65	0 (0)	0 (0)	3 (0.3)	38 (3.9)	109 (11.1)
**Digital communication tool: email**						
	20-29	1 (0.1)	12 (1.2)	24 (2.4)	60 (6.1)	62 (6.3)
	30-39	3 (0.3)	9 (0.9)	24 (2.4)	45 (4.6)	83 (8.4)
	40-49	3 (0.3)	19 (1.9)	31 (3.1)	49 (5)	98 (9.9)
	50-59	0 (0)	19 (1.9)	28 (2.8)	77 (7.8)	86 (8.7)
	60-64	1 (0.1)	14 (1.4)	14 (1.4)	33 (3.3)	41 (4.2)
	>65	0 (0)	16 (1.6)	48 (4.9)	50 (5.1)	36 (3.7)
**Digital communication tool: social networking service**						
	20-29	0 (0)	1 (0.1)	9 (0.9)	28 (2.8)	121 (12.3)
	30-39	5 (0.5)	7 (0.7)	18 (1.8)	39 (4)	95 (9.6)
	40-49	10 (1)	5 (0.5)	39 (4)	55 (5.6)	91 (9.2)
	50-59	14 (1.4)	14 (1.4)	29 (2.9)	68 (6.9)	85 (8.6)
	60-64	9 (0.9)	7 (0.7)	14 (1.4)	32 (3.2)	41 (4.2)
	>65	15 (1.5)	15 (1.5)	27 (2.7)	38 (3.9)	55 (5.6)
**Emerging technology: VR^a^, 3D printer, or AI^b^ analysis**						
	20-29	90 (9.1)	39 (4)	21 (2.1)	8 (0.8)	1 (0.1)
	30-39	107 (10.9)	23 (2.3)	23 (2.3)	8 (0.8)	3 (0.3)
	40-49	117 (11.9)	37 (3.8)	27 (2.7)	15 (1.5)	4 (0.4)
	50-59	114 (11.6)	41 (4.2)	33 (3.3)	16 (1.6)	6 (0.6)
	60-64	58 (5.9)	18 (1.8)	18 (1.8)	4 (0.4)	5 (0.5)
	>65	93 (9.4)	21 (2.1)	25 (2.5)	8 (0.8)	3 (0.3)
**Emerging technology: voice assistant (eg, Siri and Bixby)**						
	20-29	37 (3.8)	48 (4.9)	39 (4)	20 (2)	15 (1.5)
	30-39	43 (4.4)	51 (5.2)	39 (4)	25 (2.5)	6 (0.6)
	40-49	47 (4.8)	54 (5.5)	61 (6.2)	30 (3)	8 (0.8)
	50-59	64 (6.5)	58 (5.9)	57 (5.8)	21 (2.1)	10 (1)
	60-64	37 (3.8)	23 (2.3)	26 (2.6)	14 (1.4)	3 (0.3)
	>65	76 (7.7)	43 (4.4)	19 (1.9)	9 (0.9)	3 (0.3)

^a^VR: virtual reality.

^b^AI: artificial intelligence.

**Table 10:** Comparison of subset total scores by gender.

		Men (n=499), mean (SD)	Women (n=487), mean (SD)	*t* test (*df*)	*P* value
**DLS^a^ total**		66.735 (13.008)	62.834 (13.775)	4.574 (984)	<.001
	DA^b^	15.543 (5.847)	12.803 (6.158)	7.167 (984)	<.001
	CT^c^	20.764 (4.242)	19.762 (4.887)	–2.658 (984)	.001
	DE^d^	16.902 (3.173)	17.470 (3.536)	–2.658 (984)	.008
	Dcom^e^	13.527 (3.692)	12.799 (3.859)	3.029 (984)	.003
**DES^f^ total**		25.363 (5.152)	23.895 (4.713)	4.664 (984)	<.001
	Dconf^g^	14.719 (3.656)	12.669 (4.153)	8.219 (984)	<.001
	Danx^h^	10.643 (3.997)	11.226 (3.543)	–2.423 (984)	.02

^a^DLS: Digital Literacy Scale.

^b^DA: digital application.

^c^CT: critical thinking.

^d^DE: digital ethics.

^e^Dcom: digital communication.

^f^DES: Digital Efficacy Scale.

^g^Dconf: digital confidence.

^h^Danx: digital anxiety.

In addition, to examine whether there were differences in average digital efficacy across different age groups, a 1-way ANOVA was conducted (Table 11). The results indicated a statistically significant difference (*F*=18.592; *P*<.001) based on a significance level of .001. Post hoc analysis revealed that individuals in their 20s and 30s had higher digital efficacy than those in their 50s and 60s.

When examining the subfactors, it was found that individuals in their 20s and 30s scored higher than those in their 50s and 60s in the digital confidence subfactor. In addition, those in their 40s also showed higher confidence than those in their 50s and 60s. However, in the digital anxiety subfactor, the differences across age groups were not statistically significant.

**Table 11:** Comparison of subfactor and total scores.

Subfactor			Age groups (years), mean (SD)													*F* test (*df*)			*P* value			Post hoc analysis
			20s (a)		30s (b)		40s (c)		50s (d)		60-64 (e)		>65 (f)									
**DLS^a^ total**			70.415 (11.410)		68.220 (12.582)		66.180 (12.716)		62.976 (13.529)		60.369 (13.296)		58.920 (14.233)		18.076 (5)			<.001			a and b>d, e, and f; c>e and f (Scheffé test)	
	DA^b^	17.038 (5.478)		16.134 (5.917)		14.450 (6.085)		13.305 (6.010)		11.806 (5.610)		11.573 (5.812)		21.578 (5)			<.001			a>c, d, e, and f; b>d, e, and f (Scheffé test)		
	CT^c^	21.981 (3.566)		21.250 (3.819)		21.085 (4.426)		19.524 (4.657)		19.117 (5.071)		18.127 (4.969)		17.419 (5)			<.001			a and b>d, e, and f; c>e and f (Dunnett T3 test)		
	DE^d^	17.126 (2.980)		17.232 (3.181)		17.550 (3.226)		17.271 (3.418)		17.107 (3.670)		16.627 (3.801)		1.352 (5)			.24			—^e^		
	Dcom^f^	14.270 (3.688)		13.604 (3.843)		13.095 (3.578)		12.876 (3.787)		12.340 (3.701)		12.593 (3.922)		5.167 (5)			<.001			a>e and f (Scheffé test)		
**DES^g^ total**			26.478 (4.386)		26.329 (5.405)		24.830 (4.853)		24.219 (4.548)		22.738 (4.037)		22.473 (5.144)		18.592 (5)			<.001			a and b>d, e, and f; c>e and f (Scheffé test)	
	Dconf^h^	15.560 (3.093)		15.073 (3.502)		14.220 (3.915)		13.057 (3.753)		11.806 (4.118)		11.780 (4.427)		26.623 (5)			<.001			a and b>d, e, and f; c>e and f (Dunnett T3 test)		
	Danx^i^	10.918 (3.847)		11.256 (4.245)		10.610 (3.939)		11.162 (3.564)		10.932 (3.419)		10.693 (3.546)		0.802 (5)			.55			—		

^a^DLS: Digital Literacy Scale.

^b^DA: digital application.

^c^CT: critical thinking.

^d^DE: digital ethics.

^e^Not applicable.

^f^Dcom: digital communication.

^g^DES: Digital Efficacy Scale.

^h^Dconf: digital confidence.

^i^Danx: digital anxiety.

## Discussion

### Principal Findings

The detailed study findings are as follows. First, to develop a digital sensitivity scale, basic items were compiled through a literature review, and 20 items were selected after content validity was verified by 18 experts. In total, 4 factors of digital literacy and 2 factors of digital efficacy were identified. The factors of digital literacy were digital application, critical thinking, digital ethics, and digital communication, collectively accounting for 77.782% of the variance. Digital efficacy was divided into 2 factors: digital confidence and digital anxiety, showing an explanatory power of 79.378%.

The following are the specifics of the YI-DSS, which is described in this study. This scale was developed to assess digital literacy, which is known to have a significant impact on digital technology use, as well as digital efficacy, which is expected to affect intrinsic motivation and attitudes toward the use of digital technology. First, as with the Digital Literacy Scale, the domain of digital application was designed to assess the ability to create and reproduce new content based on digital technology. Next, the digital communication domain aimed to evaluate the ability to communicate and collaborate in solving problems through digital technology. The critical thinking domain was intended to assess the ability to verify the reliability and accuracy of digital information and use it critically. Finally, the digital ethics domain was designed to measure the ability to comply with ethical guidelines and understand ethical issues that arise from using digital technology. The Digital Efficacy Scale, on the other hand, was designed to assess users’ confidence and competence in using digital technology. In addition, to identify factors that impede the use of digital technologies, concerns and anxiety about the use of digital technologies were measured.

The additional analyses revealed the following findings. First, the analysis by age group confirmed a generational gap in digital literacy. In the past, when the penetration rate of digital devices was not high, the key factor contributing to the digital divide was the ease of access to digital devices. However, as the gap in physical accessibility to digital devices has been bridged, the ability to effectively use digital technology—referred to as digital literacy—has emerged as the primary cause of the digital divide [30-32].

An analysis of the types and frequency of digital device use among the respondents in this study showed that 98.2% (968/986) of the respondents frequently used smartphones. Even considering that this study was conducted through an online survey, the fact that >98% of respondents reported using their devices “frequently” or “very frequently” indicates that digital technology is used daily across all age groups, suggesting a high level of accessibility to digital devices. When analyzing the characteristics of device use by age group, >70% of respondents in their 20s, 30s, 40s, 50s, and early 60s reported frequent use of email or SNSs. Notably, the proportion of respondents in their 20s who reported frequent SNS use approached 93% (149/159, 93.7%). In addition, more than half (748/986, 75.9%) of the respondents in their late 60s reported frequent use of email and SNSs, with approximately 62% frequently using SNSs. These findings indicate that the penetration of digital devices is consistently high across all age groups and that online information activities and interactions actively take place across all ages.

Despite the improved accessibility to digital technology, there remains a clear generational gap in digital literacy and confidence in using these technologies. This finding suggests the need for age-appropriate assessments and interventions in digital sensitivity. The analysis revealed that the gap between those in their 20s and 60s was particularly pronounced. The older age groups demonstrated lower digital literacy, especially in the subfactors of digital application, practical use, and communication. Interestingly, while the frequency of online interactions for relationship building and information acquisition was high across all age groups, the generational gap in digital literacy was still evident. Older adults showed lower capabilities to verify the sources and reliability of information, critically evaluate it, and create new content and communicate on the internet. This aligns with previous research indicating that older adults, due to lower digital literacy, are more susceptible to exposure to misinformation and are at higher risk of accepting it uncritically [33-36]. Therefore, it is essential to educate and support older adults to become more active and critical consumers of information in the digital world.

Moreover, the study found significant differences in digital efficacy across age groups. The disparity in digital efficacy between individuals in their 20s and those in their 60s was particularly notable, with older adults showing lower confidence in using digital technologies. Specifically, the difference was significant in the subfactor of digital competence, indicating that older adults have lower confidence and perceived proficiency in using these technologies. However, there was no significant difference in digital anxiety, which measures concerns or fears about potential mistakes when using digital technologies. According to Bandura [37], self-efficacy increases engagement in exploratory behaviors. Individuals with high self-efficacy are more likely to take on unfamiliar tasks, actively learn and apply new information, and exhibit openness. Previous studies examining the relationship between self-efficacy and digital technology use have also found that individuals with higher self-efficacy perceive technologies as easier to use and show a higher intention to use them [30]. In this study, the analysis of the use of emerging technologies by age group revealed that, among respondents in their 20s, who exhibited the highest digital efficacy, approximately 22% (35/159) frequently used emerging technologies such as voice assistants. In contrast, only 8% (12/150) of respondents in their late 60s, which showed the lowest digital efficacy, reported frequent use of such technologies. This suggests that the level of digital efficacy not only influences the use of currently available digital technologies but also impacts the adoption of extended digital technologies, such as digital therapeutics and emerging technologies. Consequently, measuring and enhancing digital efficacy alongside digital literacy is crucial as it may affect openness to technology and proficiency and contribute to the digital divide.

Third, this study found no significant differences between age groups in ethical attitudes related to digital device use, which is one of the subfactors of digital literacy. This subfactor assesses attitudes to consider when using digital technologies, including online morality. The questions on this factor asked about awareness of clearly unethical practices, such as using illegal online routes and plagiarism, which might have led to socially desirable responses, potentially biasing the results positively. Nevertheless, measuring awareness of moral values and attitudes in digital environments remains important. According to previous research, the main factors influencing awareness of digital communication ethics are the recognition of the importance of ethical norms and individual morality [38].

As living and interacting in the digital world, which transcends the boundaries of time and space, becomes increasingly important, measuring digital ethics can serve as a guideline to help individuals align their general moral awareness, shaped by their values and beliefs, with the unique and unfamiliar environment of the digital world, thereby guiding them in focusing on and considering ethical principles.

In the current trend of digital-based medical care, the importance of digital sensitivity becomes prominent. However, despite society’s rapid digitization, there is a growing gap in the ability to accept and use digital technology.

A gap in understanding and attitude toward things appears unavoidable in a society in which the older generation, which has transitioned from an analogue or paper-based system to a digital system, coexists with the generation of “digital natives” born in a digitalized society and raised with the use of digital technology [39,40]. Therefore, it is critical to assess the extent of the digital divide and assess and improve digital literacy, a key variable that can influence the level of such a gap [39]. Studies have emphasized that successful experiences in acquiring and using ICTs during the early stages of digital literacy development are essential for applying digital technology to various contexts of life. In other words, it is necessary not only to understand how to use digital technology but also to feel competent and effective when doing so. Identifying the factors that contribute to resistance to the use of digital technology will increase digital competence and improve individual digital adaptation by increasing acceptance of and motivation for digital technology in light of such factors.

### Strengths and Limitations

In this study, we developed and validated the YI-DSS, an easy-to-use self-report tool designed to assess an individual’s digital literacy and efficacy. This study refined the concept by incorporating the latest insights related to digital literacy and included digital efficacy, which is recognized as a crucial component of digital competence. This scale is expected to enable a multidimensional assessment of an individual’s digital competence, facilitating the identification of key areas for intervention and helping bridge the information gap between individuals and generations.

The limitations of this study are as follows. First, this study aimed to create a scale to assess understanding of and competence in commonly used digital technologies. Given the rapid development of ICT equipment and digital technology in today’s society, the concept of digital literacy will continue to evolve. As a result, it will be necessary to constantly revise and supplement measurement tools to keep up with the pace of technological development.

Second, this study recruited participants and conducted the survey on the web, making it difficult to rule out the possibility that the sample was biased toward users who are more familiar with online platforms and digital devices. Future studies should consider revalidating the findings through offline or face-to-face surveys.

Finally, the survey was conducted as a one-time assessment, so test-retest reliability was not confirmed. Follow-up studies should aim to secure more in-depth reliability by conducting repeated assessments with the same group.

### Conclusions

In conclusion, this study developed and validated the YI-DSS to assess digital literacy and efficacy across various age groups. Content-valid items were analyzed using exploratory and confirmatory factor analyses, confirming a robust factor structure. The scale demonstrated strong internal consistency reliability and concurrent validity, confirming its robustness as a tool for evaluating digital literacy and efficacy. This study emphasized the importance of addressing both digital literacy and efficacy to bridge the digital divide and enhance digital adaptation across generations. While this scale provides a comprehensive tool for measuring digital literacy and efficacy, future research should focus on refining it to keep pace with technological advancements and validating the findings with more diverse populations through offline assessments.

## Abbreviations

CFI: comparative fit index
ICT: information and communications technology
KMO: Kaiser-Meyer-Olkin
RMSEA: root mean square error of approximation
SNS: social networking service
SRMR: standardized root mean square residual
TLI: Tucker-Lewis index
YI-DSS: Yongin Severance Digital Sensitivity Scale
